# State and Sociodemographic Trends in US Cigarette Smoking With Future Projections

**DOI:** 10.1001/jamanetworkopen.2025.6834

**Published:** 2025-04-25

**Authors:** Matthew D. Stone, John P. Pierce, Brian Dang, Sara B. McMenamin, Candice D. Donaldson, Xueying Zhang, David R. Strong, Yuyan Shi, Karen Messer, Dennis R. Trinidad

**Affiliations:** 1 Herbert Wertheim School of Public Health and Human Longevity Science, University of California, San Diego, La Jolla; 2 Moores Cancer Center, University of California, San Diego, La Jolla; 3 Tobacco Control Branch, California Department of Public Health, California Health and Human Services Agency, Sacramento

## Abstract

**Importance:**

There are still significant population-level health consequences of cigarette smoking in US states.

**Objective:**

To estimate whether differential sociodemographic trends in smoking prevalence since 1992 will close the prevalence gap between states by 2035.

**Design, Setting, and Participants:**

This cross-sectional study included data from 18 repeated, state-representative US Tobacco Use Supplement to the Current Population Surveys (TUS-CPS) from 1992 to 2022. Data were analyzed between June and October 2024.

**Main Outcomes and Measures:**

Nonlinear time series trends in smoking prevalence in US states from 1992 to 2022 were projected through 2035 and compared with recommended targets. States were grouped into tertiles using their 1990s prevalence. Differential change in sociodemographic groups were compared across tertiles.

**Results:**

The 18 surveys comprised 1 770 442 respondents (997 569 female [56.3%]; 146 865 Hispanic [8.3%], 160 751 non-Hispanic Black [9.1%], and 1 373 454 non-Hispanic White [78.0%]). From 1992 to 2022, all state tertiles experienced significant declines in smoking prevalence, each decreasing by approximately 13 percentage points from 2001 to 2022. In 2022, prevalence was 7.4% (95% CI, 6.9% to 7.9%) in tertile 1 (lowest 1990s prevalence), 10.0% (95% CI, 9.2% to 10.8%) in tertile 2, and 12.7% (95% CI, 11.9% to 13.4%) in tertile 3. Projections to 2035 indicated the prevalence gap closing between tertiles, to 3.8% (95% CI, 2.6% to 5.1%) in tertile 1, to 5.1% (95% CI, 3.2% to 7.2%) in tertile 2, and to 6.6% (95% CI, 4.8% to 9.1%) in tertile 3. The only sociodemographic trend that reduced the gap occurred among individuals aged 18 to 24 year (tertile 3 change, −21.3% [95% CI, −24.5% to −18.2%] vs tertile 1 change, −16.4% [95% CI, −18.5% to −14.4%]; *P* = .005). Yet age was not a significant factor in projected changes, as the decline in the group aged 50 years and older was greater in tertile 1 than 3 (−4.7% [95% CI, −5.7% to −3.8%] vs −2.3% [95% CI, −3.5% to −1.2%]; *P* < .001). By 2035, only 4 states (California, Utah, Hawaii, and Colorado) were projected to be significantly under the recommended target of 5% prevalence.

**Conclusions and Relevance:**

The findings of these repeated cross-sectional surveys suggest that the difference in cigarette smoking prevalence between historically high vs low prevalence states will shrink by 2035, primarily from much faster declines among young adults in the traditionally highest prevalence states. Slower prevalence declines among older adults are likely to slow the decline in health consequences in these states.

## Key Points

**Question:**

Will changes in smoking across sociodemographic groups remove the smoking gap between states by 2035?

**Findings:**

In this cross-sectional study of US adults, the gap identified between states that had high vs low smoking prevalence in the 1990s was projected to close before 2035. The only sociodemographic subgroup with a faster decline in the high vs low prevalence states was young adults, but this was counterbalanced by slower decline among adults aged 50 years and older.

**Meaning:**

These results suggest that while declines in prevalence among young adults promise a future smoke-free society, slower progress in the age 50 years and older group will delay the decline in US smoking-related mortality.

## Teaser

This cross-sectional study of US adults compares the prevalence of smoking by state and projects the difference between high vs low prevalence states to 2035.

## Introduction

Cigarette smoking is associated with significant health consequences, the majority of which occur in the population aged 50 years or more.^1^ Since the 1960s, the US has seen consistent, large declines in cigarette smoking prevalence,^2^ which have translated into declines in smoking-related mortality that have lagged prevalence changes by approximately 16 years.^3^ Shortly after the early scientific papers linked smoking and disease,^4,5^ the 1955 US adult smoking prevalence was 56.9%.^6^ By 2000, prevalence had halved to 23.2%,^7^ and then halved again by 2021 to 11.5%,^8^ fueling goals to end the tobacco epidemic.^9,10^ After analysis of trends, Healthy People 2030 concluded that a national smoking prevalence target below 5% was achievable.^11^

These dramatic changes have followed a variety of government initiatives focused on reducing smoking.^2^ The 2009 passage of US Family Smoking Prevention and Tobacco Control Act, which authorized the Food and Drug Administration regulation, was a key measure.^12^ However, in the US, states serve as the principal government entities for protecting public health and are also responsible for implementing major interventions such as cigarette excise taxes—a proven intervention strategy to reduce smoking prevalence.^13,14^ The first statewide tobacco control program started in California in 1988,^15,16^ and its demonstrated success^17^ was quickly followed by similar initiatives in other states,^18,19^ leading to rapid diffusion of such programs nationwide. The 1998 Master Settlement Agreement, which held the tobacco industry liability for state health care costs related to smoking diseases, restricted cigarette advertising, and provided substantial funding to states for antismoking programs as well as funding a nationwide anti-smoking campaign.^20^ Yet, from 2000 to 2023, significant differences in state spending on tobacco control and cigarette prices persisited,^21^ and these disparities were reflected in both smoking prevalence and smoking-related morbidity and mortality.^22^ Approximately one-third of US states experiences a much slower decline in both prevalence and smoking-related mortality than other states,^2^ with differences also evident by age, sex, race and ethnicity, and education.^23,24,25^

In this study, we utilized data from a large national tobacco surveillance system to report on state-level progress in reducing smoking behavior between 1992 and 2022. We tested the hypotheses that the gap in smoking prevalence between states with higher and lower smoking rates in the 1990s decreased over this time and further investigate whether these differences were associated with specific changes in sociodemographic subgroups. As changes in surveillance methods resulted in Healthy People 2030 revising their target upwards (to 6.1%),^11^ we extended the period in which state-specific prevalence projections could be reduced to 5%, to 2035. Finally, we conducted a sensitivity analysis by comparing states that our study suggests will have 2035 prevalences significantly below 5% with states that a previous publication^26^ identified as expected to meet a similar goal using the metric of state per capita cigarette sales.

## Methods

### Data Source

We utilized data from the publicly available, repeated cross-sectional Tobacco Use Supplements to the US Census Bureau’s Current Population Survey (TUS-CPS).^27^ The CPS is a large (approximately 54 000 households) monthly representative survey providing information on employment and economic well-being at both the state and national level for the civilian, noninstitutionalized US population.^28^ Since 1992, the National Cancer Institute coordinated TUS approximately every 3 years using 3 monthly samples spaced 4 months apart over 2 years and yielding approximately 150 000 respondents per wave. Response rates to the TUS-CPS ranged from a low of 62% in 2006-07 to a high of 75% in 2018-19. The CPS provides person-level supplement weights to adjust for both sampling design and nonresponse, allowing for state- and national-level estimates.^29^ For this study, all analyses were conducted between June and October 2024.

This study was conducted in accordance with the Strengthening the Reporting of Observational Studies in Epidemiology (STROBE) reporting guidelines for cross-sectional studies. The Current Population Survey (CPS) is a census survey authorized in the US Code that requires data confidentiality. The Tobacco Use Supplement surveys that are part of the CPS are overseen by the National Institutes of Health institutional review board, meet the criteria for exemption from further human participants’ protection review, and are exempt from informed consent requirements due to use of deidentified data.

### Study Outcome

On each TUS survey, respondents were asked whether they had smoked at least 100 cigarettes in their lifetime. Those who responded affirmatively were then asked if they currently smoked every day, some days, or not at all. For this analysis, individuals were classified as current smokers if they reported smoking every day or some days. Between 1992 and 2022 we obtained smoking prevalence estimates for each state for 18 different years. The TUS-CPS has used standard questions to identify respondent age, self-identified sex, educational level and race (Asian and other Pacific Islander, Black, White, and multiple races or other) and ethnicity (Hispanic or non-Hispanic).^30,31^

### Statistical Analysis

#### Estimation of Observed Prevalence in 1992 to 2022

For each survey, we calculated state-specific and national prevalence estimates with 95% confidence limits using R statistical software version 4.4.0 (R Project for Statistical Computing). All estimates were weighted by the survey weights, and confidence intervals used the published replicate weights.^27^ We use nonoverlapping confidence intervals as a conservative measure to assess statistically significant differences. As half of these US jurisdictions had 2022 populations under 5 million (and in 4 states it was under 1 million), to improve the precision of the estimates, we grouped the 51 jurisdictions into tertiles using the average smoking prevalence across surveys from 1992 to 2001.

#### Time-Series Modeling for Projection Through 2035

We used nonlinear approaches to project the prevalence data using both autoregressive integrated moving average (ARIMA) and exponential smoothing (ETS) algorithms. An ARIMA model has 3 components that model the autocorrelation in the data: an autoregressive component (p), a preprocessing component which differences the data (d), and an error term identified from the moving average (q). ARIMA models were fitted using the fable package in R.^32^ This algorithm combines unit root tests to determine the value of d, followed by minimization of the sample size–adjusted Akaike Information Criterion (AIC)^33^ to select values of p and q. To select the best ARIMA model, we allowed for potential inclusion of a “drift” constant and conducted a full neighborhood search without approximation. Data were analyzed on the level of the population by using aggerated prevalence estimates.

Exponential smoothing applies exponentially decaying weights so recent observations influence forecasts more.^34^ Exponential smoothing has the 3 main components (error, trend, season) that are each able to be modeled in 3 different ways (additive, multiplicative, and none or not included). ETS models were fitted using the ETS function from the fable package. The choice to use an ARIMA or ETS model was determined by the lowest AIC.

#### Imputation of Missing Values

The TUS-CPS provides time series data, in which there is temporal dependence between individual estimates. Standard forecasting approaches for time series have an assumption that observations are spaced at equal intervals^35^ so that missing observations need to be imputed. To impute these, we used linear spline interpolation, using the na_interpolation function from the imputeTS package.

#### Assessment of State Differences

The 50 US states and the District of Columbia were categorized into tertiles based on average smoking prevalence across the surveys prior to the 2 decades ending in 2022 (ie, 1992, 1993, 1995, 1996, 1998, 1999, and 2001 surveys). Smoking prevalence for each tertile was then plotted through 2022 and projected through 2035 with 95% CIs.

In addition to our primary analysis, we conducted a sensitivity analysis to compare states projected to have 2035 smoking prevalences significantly below 5% with those previously identified as meeting a similar target based on state per capita cigarette sales. This additional analysis was designed to evaluate the robustness of our projections across different tobacco consumption metrics and to account for potential discrepancies due to state-specific factors, such as policy variations and market behaviors. Data for this analysis were obtained from Leas et al,^26^ which utilized state-level cigarette sales data compiled from the 1950-2020 Tax Burden on Tobacco Reports as an independent indicator of tobacco consumption. We then applied our projection methodology to these sales data to assess the consistency of our prevalence-based findings with an alternative metric.

#### Sociodemographic Changes in Prevalence

Using separate survey-weighted logistic regression analyses (with the survey package), we examined how the prevalence changes between 2001-2002 to 2018-2022 differed across tertiles for the same sociodemographic subgroups. For each tertile, we calculated the prevalence difference over time by sociodemographic subgroup and then assessed whether the change within sociodemographic subgroup was different between tertiles 1 and 3. We conducted omnibus tests of 3-way interactions using likelihood ratio tests to assess the overall significance of interactions among tertile, survey year, and sociodemographic characteristics. Post hoc pairwise comparisons with multivariate t adjustments were performed using the emmeans package.

Race and ethnicity were considered as variables in this study because extant research has demonstrated significant disparities in smoking prevalence across different racial and ethnic groups. Categories for race included Asian and other Pacific Islander, Black, White, and multiple races or other (American Indian and Alaska Native, Native Hawaiian or Pacific Islander) with or without Hispanic ethnicity.

## Results

A total of 1 770 442 respondents were analyzed. Unweighted, the sample comprised 997 569 female respondents (56.3%); 160 751 identified as non-Hispanic Black (9.1%), 146 865 as Hispanic (8.3%), and 1 373 454 as non-Hispanic White (78.0%). The largest cohort by age was 35 to 49 years (29.6%); 961 968 (54.3%) had attained some college education.

### State Prevalence Estimates for the 1990s, 2022, and projected to 2035

The average national prevalence from 1992-2001 was 22.8% (95% CI, 22.6% to 22.9%) and there was a large difference in smoking prevalence across the 50 US states plus the DC ranged from 14.8% in Utah to 30.6% in Kentucky (Table 1). By 2022, the national prevalence estimate was 9.4% (95% CI, 9.0% to 9.7%) and while prevalence had significantly declined in all states, large state differences persisted, from a low of 1.4% (95% CI, 0.4% to 3.0%) in Hawaii to 16.0% (95% CI, 10.9%-23.3%) in Iowa.

**Table 1.** State-Level Smoking Prevalence Prior to 2001 and in 2022 and Projections to 2035

State	2022 Population, million people^a^	Weighted prevalence (95% CI)			
		Observed		Projected	
		1992-2001	2022	2035 Projection^b^	Algorithm selected^c^
Overall US	333.2	22.8 (22.6-22.9)	9.4 (9.0-9.7)	4.9 (3.5-6.6)	ARIMA
**Tertile 1**					
Tertile mean	168.7	20.5 (20.3-20.7)	7.4 (6.9-7.9)	3.8 (2.6-5.1)	ARIMA
Utah	3.4	14.8 (13.4-16.3)^d^	4.4 (2.0-6.7)^d^	2.5 (1.4-4.0)^d^^,^^e^	ARIMA
California	39.0	17.6 (17.1-18.1)^d^	5.6 (4.7-6.5)^d^	3.3 (2.4-4.3)	ETS
Hawaii	1.4	19.8 (18.6-20.8)^d^	6.6 (4.2-9.0)^d^	1.4 (0.4-3.0)^d^	ETS
Massachusetts	7.0	19.8 (19.0-20.6)^d^	8.3 (6.2-10.5)	5.6 (3.6-7.6)	ARIMA
Maryland	6.2	20.8 (19.5-22.0)^d^	8.0 (5.4-10.7)	5.2 (4.1-6.9)	ARIMA
Connecticut	3.6	20.8 (19.2-22.4)^d^	6.5 (3.5-9.5)	3.8 (2.5-5.7)	ARIMA
New Jersey	9.3	20.9 (20.0-21.7)^d^	6.0 (4.2-7.8)^d^	3.5 (2.0-6.6)	ARIMA
Arizona	7.4	21.3 (20.1-22.6)^d^	6.7 (4.8-8.5)^d^	3.8 (2.2-6.2)	ARIMA
New York	19.7	21.5 (21.0-22.1)^d^	8.1 (6.3-9.8)	5.0 (3.1-6.7)	ETS
Oregon	4.2	21.6 (20.2-22.9)	8.4 (5.3-11.5)	4.1 (2.1-6.9)	ETS
Florida	22.2	22.1 (21.5-22.7)	8.4 (6.7-10.1)	5.4 (4.7-6.3)	ETS
Colorado	5.8	22.2 (21.2-23.2)	5.9 (3.2-8.6)^d^	1.5 (0.4-4.1)	ETS
Texas	30.0	22.3 (21.6-22.9)	9.0 (7.7-10.4)	5.7 (3.6-8.0)	ARIMA
Rhode Island	1.1	22.3 (21.1-23.4)	7.5 (4.3-10.8)	4.8 (2.4-8.9)	ARIMA
Idaho	1.9	22.3 (20.6-24.0)	7.5 (5.0-10.1)	5.7 (4.2-7.9)	ARIMA
District of Columbia	0.7	22.4 (21.3-23.5)	8.2 (5.9-10.5)	5.4 (3.2-7.9)	ARIMA
Minnesota	5.7	22.4 (21.4-23.5)	9.1 (6.3-11.9)	4.0 (2.8-5.5)	ETS
**Tertile 2**					
Tertile mean	81.7	23.5 (23.2-23.7)	10.0 (9.2-10.8)	5.1 (3.2-7.2)	ARIMA
Nebraska	2.0	22.4 (21.4-23.5)	10.8 (8.4-13.2)	6.6 (4.5-9.4)	ETS
Georgia	10.9	22.5 (21.5-23.5)	7.0 (4.9-9.2)	4.3 (2.1-7.3)	ARIMA
North Dakota	0.8	22.6 (21.0-24.2)	10.1 (7.1-3.1)	7.3 (4.1-12.1)	ARIMA
New Mexico	2.1	22.7 (21.5-24.0)	9.4 (6.3-12.5)	6.3 (4.1-8.7)	ARIMA
Washington	7.8	22.8 (21.5-24.1)	10.3 (7.0-13.9)	7.1 (4.9-9.7)	ETS
Iowa	3.2	22.9 (21.3-24.5)	15.8 (11.6-20.0)^e^	16.0 (10.9-23.3)^e^	ETS
Montana	1.1	23.5 (21.9-25.0)	13.3 (10.1-16.6)^e^	10.6 (6.9-14.4)^e^	ARIMA
Pennsylvania	13.0	23.6 (23.0-24.3)	10.3 (8.0-12.5)	7.0 (5.3-9.1)	ARIMA
Virginia	8.7	23.7 (22.8-24.5)	7.8 (5.6-10.1)	5.0 (3.5-6.6)	ARIMA
New Hampshire	1.4	23.9 (22.6-25.2)	11.0 (7.5-14.4)	8.0 (4.3-14.8)	ARIMA
South Dakota	0.9	23.9 (22.6-25.3)	13.8 (10.9-16.7)^e^	10.9 (7.1-15.2)^e^	ARIMA
Alabama	5.1	24.0 (22.7-25.2)	11.1 (7.5-14.6)	7.8 (3.8-14.4)	ARIMA
Illinois	12.6	24.0 (23.3-24.6)	9.5 (7.5-11.6)	6.3 (4.5-8.0)	ARIMA
Kansas	2.9	24.0 (22.7-25.3)	12.4 (8.4-16.4)	9.3 (6.1-13.7)	ARIMA
Mississippi	2.9	24.0 (22.1-25.9)	14.3 (11.3-17.2)^e^	12.4 (10.8-15.8)^e^	ETS
South Carolina	5.3	24.2 (23.0-25.3)	11.5 (8.5-14.5)	8.3 (4.9-14.0)	ARIMA
Delaware	1.0	24.2 (22.8-25.7)^e^	10.1 (6.7-13.5)	7.1 (4.9-9.7)	ARIMA
**Tertile 3**					
Tertile mean	82.8	26.3 (26.0-26.6)	12.7 (11.9-13.4)	6.6 (4.8-9.1)	ARIMA
Louisiana	4.6	24.4 (23.3-25.5)^e^	13.7 (11.2-16.3)^e^	8.4 (6.4-10.7)	ETS
Vermont	0.7	24.8 (23.4-26.2)^e^	9.5 (6.9-12.1)	6.2 (4.0-8.6)	ETS
Wyoming	0.6	25.1 (23.9-26.3)^e^	13.5 (9.0-17.9)	14.0 (7.8-23.7)^e^	ETS
Missouri	6.2	25.2 (23.7-26.7)^e^	12.2 (8.6-15.9)	9.3 (5.6-14.6)	ARIMA
Wisconsin	5.9	25.3 (23.8-26.8)^e^	12.7 (10.5-14.8)^e^	9.6 (5.9-13.3)	ARIMA
Maine	1.3	25.3 (23.7-27.0)^e^	16.4 (10.8-22.0)^e^	12.7 (8.6-17.2)^e^	ARIMA
Michigan	10.0	25.6 (24.7-26.5)^e^	12.4 (10.0-14.8)^e^	7.0 (6.6-9.5)	ETS
Ohio	11.8	25.8 (25.0-26.5)^e^	12.9 (10.9-15.0)^e^	8.3 (5.9-11.1)	ARIMA
North Carolina	10.7	25.8 (24.9-26.7)^e^	11.7 (9.2-14.2)	8.2 (5.8-11.2)	ARIMA
Nevada	3.2	26.0 (24.5-27.4)^e^	7.7 (5.1-10.3)	4.9 (2.3-8.4)	ETS
Oklahoma	4.0	26.6 (25.3-27.9)^e^	14.6 (10.3-18.9)^e^	15.1 (8.1-23.7)^e^	ETS
Alaska	0.7	26.8 (24.8-28.7)^e^	13.4 (9.9-17.2)^e^	9.8 (7.1-13.5)^e^	ARIMA
Arkansas	3.0	27.2 (26.1-28.3)^e^	12.3 (9.2-15.5)	8.6 (6.0-12.4)	ARIMA
Tennessee	7.1	27.2 (25.7-28.7)^e^	13.5 (11.2-15.7)^e^	10.1 (7.6-13.2)^e^	ETS
Indiana	6.8	27.2 (25.8-28.7)^e^	12.3 (9.6-14.9)	10.0 (7.8-12.6)^e^	ARIMA
West Virginia	1.8	28.7 (27.0-30.3)^e^	15.9 (12.8-19.1)^e^	13.9 (11.2-16.5)^e^	ARIMA
Kentucky	4.5	30.6 (29.4-31.9)^e^	13.3 (10.0-16.6)^e^	8.8 (6.2-12.1)	ARIMA

Abbreviations: ARIMA, autoregressive integrated moving average; ETS, exponential smoothing.

^a^ Resident population of the US in 2022.

^b^ Detailed projections for each state are presented in the eFigure in Supplement 1.

^c^ Information on the selection of models is in the eTable in Supplement 1.

^d^ Estimates with upper confidence intervals below the lower confidence interval for the overall US.

^e^ Estimates with lower confidence intervals above the upper confidence interval for the overall US.

Mean prevalence estimates between tertiles (tertile 1, 20.5% [95% CI, 20.3% to 20.7%]; tertile 2, 23.5% [95% CI, 23.2% to 23.7%]; tertile 3, 26.3% [95% CI, 26.0% to 26.6%]) had nonoverlapping confidence intervals. Nine of the 17 jurisdictions in tertile 1 had prevalence estimates with confidence intervals below the US average, and none of the states in the other tertiles (denoted in Table 1). Conversely, all of the states in tertile 3 and 1 in tertile 2 had prevalence estimates with confidence intervals above the US average.

By 2022, confidence intervals still did not overlap between the tertiles (prevalence estimates: tertile 1, 7.4% [95% CI, 6.9% to 7.9%]; tertile 2, 10.0% [95% CI, 9.2% to 10.8%]; tertile 3, 12.7% [95% CI, 11.9% to 13.4%]). The number of states with a prevalence significantly below the US average had been reduced from 9 to 6 and the number of states with a prevalence significantly higher than the US average had been reduced from 18 to 14.

The parameters for selecting the optimal model (ARIMA or ETS) to fit each state’s data are presented in eTable in Supplement 1 and the detailed projections to obtain each state and tertile’s expected prevalence in 2035 are presented in eFigure in Supplement 1. Using these models, we project that, in 2035, the national smoking prevalence will be 4.9% (95% CI, 3.5% to 6.6%) and the prevalence estimates for each tertile (tertile 1, 3.8% [95% CI, 2.6% to 5.1%]; tertile 2, 5.1% [95% CI, 3.2% to 7.2%]; tertile 3, 6.6% [95% CI, 4.8% to 9.1%]) will be similar. Compared with the projected US mean prevalence, 1 state (Hawaii) would be significantly below and 11 states (all smaller population states) significantly above. In 3 of these higher prevalence states (Iowa, Wyoming, Oklahoma), the preferred model had a flat line projection from the 2022 prevalence with wide confidence intervals.

### Color-Coded US State Maps Showing Changing Cigarette Prevalence From 1992 to 2035

**Figure 1.**
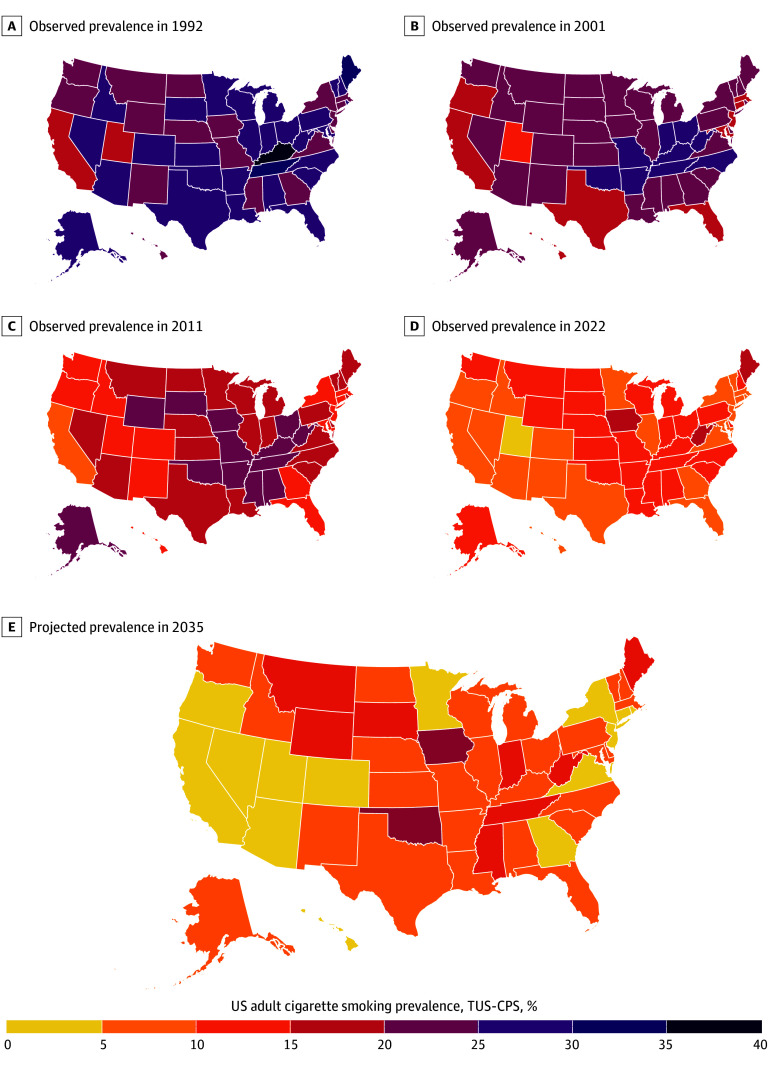
Color-Coded US State Maps of Cigarette Smoking Prevalence at 5 Time Points Since 1992 Model estimated observed prevalence for each US state from the Tobacco Use Supplement to the Current Population Survey (TUS-CPS) surveillance system 1992-2022 with estimated prevalence in 2035 from projections using the best fit model of the observed data.

We plotted the state prevalence at approximate decade intervals on US maps in Figure 1. In 1992, the majority of US states had prevalences above 20%. With each decade, cigarette use became less prevalent in almost every state, so that by 2022 states were mainly below 10%. Projected data shows a further progression to reduced prevalence in nearly all states with the western region mainly having a prevalence below 5%. Thus, while the decline in prevalence may have been led initially by a few states, it quickly spread across all US states.

### Prevalence Trends and Projections With States Grouped Into Tertiles

**Figure 2.**
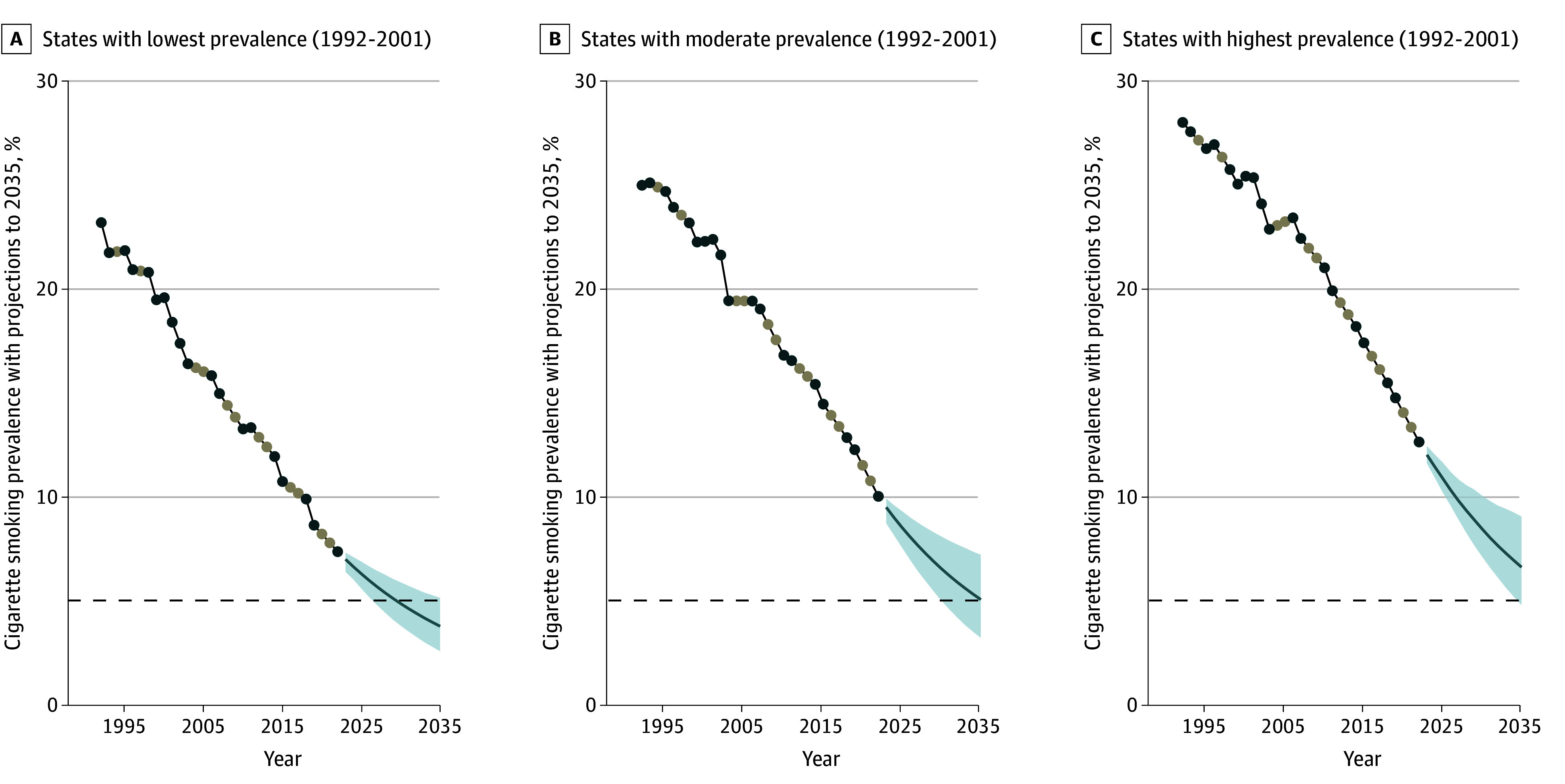
Trends in Cigarette Smoking Prevalence Among US States Divided Into Tertiles on Average Prevalence, 1992-2001 States are divided into tertiles on their average prevalence across 1992-2001 Tobacco Use Supplement to the Current Population Survey (TUS-CPS) estimates. Blue dots are observed data from surveys, grey dots are imputed data (time series analyses require imputation of missing values). The autoregressive integrated moving average (ARIMA) model was selected as the best fit using Akaike Information Criteria. The blue line (without dots) is the projection to 2035 and the blue shaded area around the line is the 95% confidence interval. The dotted line is the 5% target prevalence.

Grouping the states into large population tertiles resulted in a minimum 2022 population with each tertile above 80 million people, which resulted in good precision for the 2035 estimates (Figure 2). These tertiles had similar declines between 2001 and 2022 (tertile 1, 13.1 percentage points [pp]; tertile 2, 13.5 pp; and tertile 3, 13.6 pp). However, the different patterns of change over the 2 decades resulted in projections with differential rates of decline through the period (tertile 1, −0.28 pp per year; tertile 2, −0.38 pp per year; tertile 3, −0.47 pp per year). This suggests that current trends will result in a closing of the prevalence gap across tertiles by 2035.

### Did State Tertiles Close Any Sociodemographic Gaps in Prevalence Between 2001-2022?

In both the highest and lowest tertiles, between 2001-2002 and 2018-2022, the highest prevalence decline occurred among the 18-to-24-year-old population, but it was much faster in tertile 3 than in 1 (−21.3% [95% CI, −24.5% to −18.2%] vs −16.4% [95% CI, −18.5% to −14.4%]; *P* = .005) (Table 2). However, there was no overall age effect (omnibus likelihood ratio *P* = .66), as the much faster decline in tertile 3 18-to-24-year-olds was counteracted by a decline that was half the rate of tertile 1 among the much larger age 50 years and older population (−2.3% [95% CI, −3.5% to −1.2%] vs −4.7% [95% CI, −5.7% to −3.8%]; *P* < .001). The omnibus likelihood ratio test showed no difference in the prevalence decline between tertiles 1 and 3 for sex, race and ethnicity, or education level achieved. Nor were there any significant differences in the declines within any subgroups of these variables. Thus, prevalence differences between subgroups of these sociodemographic variables are likely to remain in 2035.

**Table 2.** Sociodemographic Differences in 2002 to 2022 Cigarette Smoking Prevalence Change Between State Tertile 1 and 3^a^

Sociodemographic characteristics	State tertile 1		State tertile 3		Difference in declines T1 vs T3, *P* value^b^	Omnibus Test^c^
	2001-02 Prevalence, mean % (95% CI)	Prevalence difference by 2018-22, % difference (95% CI)	2001-02 Prevalence, mean % (95% CI)	Prevalence difference by 2018-22, % difference (95% CI)		
Age						
18-24 y	21.5 (20.3 to 22.7)	−16.4 (−18.5 to −14.4)	30.9 (29.1 to 32.9)	−21.3 (−24.5 to −18.2)	.005	.66
25-34 y	19.4 (18.6 to 20.3)	−10.9 (−12.4 to −9.4)	27.0 (25.7 to 28.3)	−10.0 (−12.5 to −7.5)	.98	
35-49 y	21.3 (20.5 to 22.2)	−11.9 (−13.3 to −10.5)	29.7 (28.7 to 30.7)	−12.1 (−14.0 to −10.1)	>.99	
≥50 y	13.9 (13.4 to 14.5)	−4.7 (−5.7 to −3.8)	16.7 (16.1 to 17.3)	−2.3 (−3.5 to −1.2)	<.001	
Sex						
Female	16.0 (15.6 to 16.4)	−8.6 (−9.2 to −7.9)	22.7 (22.0 to 23.4)	−9.1 (−10.2 to −8.0)	.68	.95
Male	20.7 (20.1 to 21.4)	−10.7 (−11.7 to −9.7)	26.6 (25.7 to 27.5)	−10.0 (−11.5 to −8.6)	.75	
Race and ethnicity						
Hispanic	14.7 (13.6 to 15.8)	−7.9 (−9.7 to −6.1)	21.4 (18.5 to 24.7)	−11.3 (−16.4 to −6.2)	.72	.38
Non-Hispanic Asian or Pacific Islander	11.8 (10.6 to 13.1)	−6.7 (−8.7 to −4.7)	13.5 (10.6 to 17.2)	−7.8 (−13.1 to −2.4)	>.99	
Non-Hispanic Black	18.5 (17.2 to 19.8)	−9.2 (−11.4 to −7.0)	24.8 (23.3 to 26.4)	−8.5 (−11.4 to −5.6)	>.99	
Non-Hispanic White	19.6 (19.1 to 20.2)	−9.8 (−10.8 to −8.9)	24.5 (23.9 to 25.2)	−9.4 (−10.6 to −8.2)	>.99	
Non-Hispanic other or multiple	25.1 (19.9 to 31.6)	−11.6 (−20.4 to −2.9)	42.1 (38.2 to 46.3)	−18.1 (−25.7 to −10.5)	.78	
Highest education						
Less than high school	21.9 (20.8 to 23.1)	−8.5 (−10.8 to −6.3)	32.0 (30.6 to 33.5)	−5.9 (−9.2 to −2.6)	.65	.83
High school	23.4 (22.6 to 24.3)	−10.8 (−12.2 to −9.3)	30.2 (29.3 to 31.2)	−9.6 (−11.4 to −7.9)	.84	
Some college	19.9 (19.0 to 20.9)	−9.7 (−11.4 to −8.0)	25.1 (24.1 to 26.2)	−8.8 (−10.7 to −6.8)	.98	
College graduate	11.8 (11.3 to 12.3)	−6.6 (−7.4 to −5.9)	13.4 (12.7 to 14.1)	−5.6 (−6.9 to −4.4)	.54	

^a^ Parameter estimates from separate weighted logistic regressions projecting cigarette smoking prevalence including a year × state tertile × sociodemographic interaction term.

^b^ The interaction with post-hoc pairwise contrasts utilized a multivariate t adjustment.

^c^ Omnibus test for an overall difference across the groups.

### Sensitivity Analysis for Comparing Achievement of Target Smoking Level by 2035 From Prevalence vs State Per Capita Cigarette Sales

From Table 1, in 2035, only 4 states (California, Utah, Colorado, and Hawaii) are projected to have a prevalence that will be significantly below the 5% target level. The previously published projections^26^ based on state sales (cigarette packs per capita), also identified 4 states (Utah, California, New York, Washington) with a projected 2035 sales below a target of 13 packs per capita, which they showed approximated the target 5% prevalence. Two states (Utah, California) are projected to be significantly below both the prevalence and the sales targets (Table 3). The additional 2 states (Hawaii, Colorado) that we project to be below the prevalence target were projected to be significantly above the sales target. Both New York and Washington were projected to be well below the sales target but unlikely to be significantly below the 5% prevalence target.

**Table 3.** Comparison of States Projected to Meet Cigarette Targets for Prevalence and/or State Pack Sales

States projections on both prevalence and cigarette pack sales	US states	2035 Projected prevalence		Projected median state sales^a^	
		2035 Projection	2035 Probability prevalence <5%	2035 Median cigarette sales per capita	2035 Probability sales <13 ppc
States projected to be significantly below target on both prevalence and sales	Utah	2.5 (1.4-4.0)	>0.95	7.2 (5.0-9.7)	>0.95
	California	3.3 (2.4-4.3)	>0.95	6.8 (4.6-9.0)	>0.95
States projected to be significantly below target on prevalence but not sales	Hawaii	1.4 (0.4-3.0)	>0.95	23.1 (6.3-79.4)	0.20
	Colorado	1.5 (0.4-4.1)	>0.95	15.2 (12.4-18.6)	0.11
States projected to be significantly below target on sales but not prevalence	New York	5.0 (3.1-6.7)	0.50	3.8 (1.9-6.3)	>0.95
	Washington	7.1 (4.9-9.7)	<0.10	5.8 (3.8-8.8)	>0.95

Abbreviation: ppc, packs per capita.

^a^ Projections of per capita sales from Leas et al.^26^

## Discussion

In this repeated cross-sectional study of US state- and national-level smoking trends, we found that large differences in smoking prevalence existed across US states in both the 1990s and in 2022, although our projections indicated that these differences are expected to diminish considerably by 2035, with national prevalence approaching 5%. However, differences in prevalence across age, sex, race and ethnicity, and education persisted. We observed that the most consequential narrowing of the prevalence gap occurred in states with historically high smoking rates, which experienced a significantly larger decline in young adult prevalence compared with states with lower historical rates. This gain was offset by a significantly slower decline among the age 50 years and older population—likely reflecting lower successful cessation in this age group^36^—which we anticipate would delay the reduction in smoking-related mortality in those states.

Furthermore, this study’s 2022 US smoking prevalence estimates, based on the TUS-CPS surveillance system, was significantly lower than that reported by NHIS,^37^ consistent with previous findings that have noted a roughly 10% higher prevalence in NHIS estimates.^2,37^ Although both high-quality surveillance systems used the same questions to assess smoking prevalence, methodological differences—such as TUS-CPS’ design for state and national estimates vs NHIS’s focus on national estimates—likely contributed to these discrepancies.

Finally, our study’s projections indicated that only 4 US states would likely achieve a prevalence significantly below 5% by 2035, mirroring targets estimated from cigarette sales data.^26^ However, only 2 states (Utah and California) were projected to be below the target in both studies. In contrast, 2 states (Hawaii and Colorado) were projected to be below the target based on prevalence but not on state cigarette sales, a discrepancy that may arise when a small state with a low smoking prevalence, like Hawaii,^38^ is geographically isolated and experiences substantial tourism. Conversely, while New York and Washington were expected to meet the target based on state cigarette sales, they are not anticipated to meet the prevalence target—a finding that could be explained by high cigarette taxes, which resident smokers avoid or evade by purchasing cigarettes elsewhere.^39^ For instance, in 2013, a Virginia State Crime Commission^40^ documented that New York had a significant black market for cigarettes that had paid the much lower Virginia state taxes.

### Strengths and Limitations

A strength of this study is its use of a major US state and nationally representative surveillance system with over 30 years of consistent tracking of cigarette smoking. However, this surveillance system does not provide estimates annually, necessitating imputation for missing years to assess nonlinear trends. Another limitation is that, although each survey achieved a relatively high response rate, our statistical adjustments (including imputation) cannot eliminate the impact of missing data on our estimates. While our imputation methods help mitigate the effects of missing values, they may not fully capture the true variability or potential biases present during periods with incomplete data. A sensitivity analysis further indicated that a similar proportion of states were classified as meeting the 2035 target when using alternative measures of state cigarette smoking, suggesting that the influence of missing data on our overall projections is modest but cannot be completely ruled out.

## Conclusions

The rapid decline in US cigarette smoking prevalence is occurring across all US states, and the findings of this study suggest that the major differences across US states are disappearing. However, between 2001 and 2022, there was no evidence that this closing of the gap between historically high or low prevalence states was associated with different rates of change by sex, race and ethnicity, or education. The highest prevalence states had much larger prevalence declines among young adults which are expected to affect prevalence rates in the longer term. However, these changes were counterbalanced by much slower prevalence declines among the age 50 years and older population. This effect is likely to delay the time lag between a decline in prevalence and a reduction in the health consequences of smoking in these historically high prevalence states.

## Notes

Supplement 1. **eFigure.** Individual State Models With Projections for States **eTable.** Parameters Used for Selecting Each State Model With Best ﬁt to Observed Data

Supplement 2.
Data Sharing Statement

## References

[1] US Office of the Surgeon General. Smoking and health: report of the advisory committee to the Surgeon General of the Public Health Service. PHS Publication No. 1103. 1964. Accessed October 29, 2024. https://profiles.nlm.nih.gov/101584932X202

[2] National Center for Chronic Disease Prevention and Health Promotion (US) Office on Smoking and Health. The health consequences of smoking—50 years of progress: a report of the Surgeon General. 2014. Accessed October 29, 2024. https://www.ncbi.nlm.nih.gov/books/NBK179276/

[3] Pierce JP, Messer K, White MM, Kealey S, Cowling DW. Forty years of faster decline in cigarette smoking in California explains current lower lung cancer rates. Cancer Epidemiol Biomarkers Prev. 2010;19(11):2801-2810. doi:10.1158/1055-9965.EPI-10-0563 20852009

[4] Doll R, Hill AB. Smoking and carcinoma of the lung: preliminary report. BMJ. 1950;2(4682):739-748. 14772469 10.1136/bmj.2.4682.739 PMC2038856

[5] Wynder EL, Graham EA. Tobacco smoking as a possible etiologic factor in bronchiogenic carcinoma; a study of 684 proved cases. JAMA. 1950;143(4):329-336. doi:10.1001/jama.1950.02910390001001 15415260

[6] Ahmed PI, Gleeson GA. Changes in Cigarette Smoking Habits between 1955 and 1966. National Health Survey Vital Health and Statistics, Series 10 No, 59, April 1970. Accessed October 29, 2024. https://stacks.cdc.gov/view/cdc/12522/cdc_12522_DS1.pdf 5310201

[7] Trosclair HC, Pederson L. Cigarette smoking among adults—United States, 2000. MMWR. 2002;51(29):642-645.

[8] Cornelius ME, Loretan CG, Jamal A, . Tobacco product use among adults—United States, 2021. MMWR Morb Mortal Wkly Rep. 2023;72(18):475-483. doi:10.15585/mmwr.mm7218a1 37141154 PMC10168602

[9] Thomson G, Wilson N, Blakely T, Edwards R. Ending appreciable tobacco use in a nation: using a sinking lid on supply. Tob Control. 2010;19(5):431-435. doi:10.1136/tc.2010.036681 20876079

[10] Malone RE. Imagining things otherwise: new endgame ideas for tobacco control. Tob Control. 2010;19(5):349-350. doi:10.1136/tc.2010.039727 20876073

[11] United States Department of Health and Human Services. Reduce current cigarette smoking in adults—TU-02. Healthy People 2030. Accessed October 29, 2024. https://health.gov/healthypeople/objectives-and-data/browse-objectives/tobacco-use/reduce-current-cigarette-smoking-adults-tu-02

[12] Husten CG, Deyton LR. Understanding the Tobacco Control Act: efforts by the US Food and Drug Administration to make tobacco-related morbidity and mortality part of the USA’s past, not its future. Lancet. 2013;381(9877):1570-1580. doi:10.1016/S0140-6736(13)60735-7 23642698

[13] Farrelly MC, Bray JW; Centers for Disease Control and Prevention (CDC). Response to increases in cigarette prices by race/ethnicity, income, and age groups–United States, 1976-1993. MMWR Morb Mortal Wkly Rep. 1998;47(29):605-609. 9699809

[14] Chaloupka FJ, Straif K, Leon ME. Effectiveness of tax and price policies for tobacco control. Tob Control. 2011;14:235-238. doi:10.1136/tc.2010.039982 21115556

[15] Bal DG, Kizer KW, Felten PG, Mozar HN, Niemeyer D. Reducing tobacco consumption in California—development of a statewide anti-tobacco use campaign. JAMA. 1990;264(12):1570-1574. doi:10.1001/jama.1990.03450120082034 2395199

[16] Roeseler A, Burns D. The quarter that changed the world. Tob Control. 2010;19(Suppl_1)(suppl 1):i3-i15. doi:10.1136/tc.2009.030809 20382647 PMC2976491

[17] Pierce JP, Gilpin EA, Emery SL, . Has the California tobacco control program reduced smoking? JAMA. 1998;280(10):893-899. doi:10.1001/jama.280.10.893 9739973

[18] Biener L, Harris JE, Hamilton W. Impact of the Massachusetts tobacco control programme: population based trend analysis. BMJ. 2000;321(7257):351-354. doi:10.1136/bmj.321.7257.351 10926595 PMC27453

[19] Farrelly MC, Pechacek TF, Chaloupka FJ. The impact of tobacco control program expenditures on aggregate cigarette sales: 1981-2000. J Health Econ. 2003;22(5):843-859. doi:10.1016/S0167-6296(03)00057-2 12946462

[20] Daynard RA, Parmet W, Kelder G, Davidson P. Implications for tobacco control of the multistate tobacco settlement. Am J Public Health. 2001;91(12):1967-1971. doi:10.2105/AJPH.91.12.1967 11726376 PMC1446915

[21] Messer K, Pierce JP, Chen J, . Cigarette smoking decline among US young adults from 2000 to 2019, in relation to state-level cigarette price and tobacco control expenditure. Tob Control. Published online July 9, 2024. doi:10.1136/tc-2023-058483 38981671 PMC12175774

[22] Pierce JP, Shi Y, McMenamin SB, . Trends in lung cancer and cigarette smoking: California compared to the rest of the United States. Cancer Prev Res (Phila). 2019;12(1):3-12. doi:10.1158/1940-6207.CAPR-18-0341 30305281 PMC7389269

[23] Jeon J, Inoue-Choi M, Mok Y, . Mortality relative risks by smoking, race/ethnicity, and education. Am J Prev Med. 2023;64(4)(suppl 1):S53-S62. doi:10.1016/j.amepre.2022.12.006 36775754 PMC11186465

[24] Thomson B, Emberson J, Lacey B, . Association between smoking, smoking cessation, and mortality by race, ethnicity, and sex among US adults. JAMA Netw Open. 2022;5(10):e2231480. doi:10.1001/jamanetworkopen.2022.31480 36279139 PMC9593233

[25] Choi K, Jones JT, Ruybal AL, McNeel TS, Duarte DA, Webb Hooper M. Trends in education-related smoking disparities among U.S. Black or African American and White adults: intersections of race, sex, and region. Nicotine Tob Res. 2023;25(4):718-728. doi:10.1093/ntr/ntac238 36239224 PMC10032197

[26] Leas EC, Trinidad DR, Pierce JP, McMenamin SB, Messer K. Trends in cigarette consumption across the United States, with projections to 2035. PLoS One. 2023;18(3):e0282893. doi:10.1371/journal.pone.0282893 36913367 PMC10010542

[27] National Cancer Institute. The tobacco use supplement to the current population survey. Last updated May 2023. Accessed October 29, 2024. https://cancercontrol.cancer.gov/brp/tcrb/tus-cps

[28] US Bureau of the Census. Current population survey methodology. Revised June 4, 2024. Accessed October 29, 2024. https://www.census.gov/programs-surveys/cps/technical-documentation/methodology.html

[29] Liu B. Fay’s variance estimation method for combining multiple TUS-CPS data. Surveillance Research Program, National Cancer Institute technical report No. 2020-1. Published online 2020. Accessed October 29, 2024. https://surveillance.cancer.gov/reports/tech2020.01.pdf

[30] Sakuma KK, Pierce JP, Fagan P, . Racial/ethnic disparities across indicators of cigarette smoking in the era of increased tobacco control, 1992-2019. Nicotine Tob Res. 2021;23(6):909-919. doi:10.1093/ntr/ntaa231 33196799 PMC8522466

[31] Cao P, Jeon J, Tam J, . Smoking disparities by level of educational attainment and birth cohort in the U.S. Am J Prev Med. 2023;64(4)(suppl 1):S22-S31. doi:10.1016/j.amepre.2022.06.021 36935129 PMC10177656

[32] Hyndman RJ, Khandakar Y. Automatic time series forecasting: the forecast package for R. J Stat Softw. 2008;27:1-22. doi:10.18637/jss.v000.i00

[33] Cavanagh JE, Neath A. The Akaike information criterion: Background, derivation, properties, application, interpretation and refinements. Wiley Interdiscip Rev Comput Stat. 2019;11(3):e1460. doi:10.1002/wics.1460

[34] Shastri S, Sharma A, Mansotra V, Sharma A, Bhadwal A, Kumari M. A study on exponential smoothing method for forecasting. Int J Comput Sci Eng. 2018;6:482-485. doi:10.26438/ijcse/v6i4.482485

[35] Box GEP, Jenkins GM, Ljung GM. Time Series Analysis: Forecasting and Control. 5th ed. John Wiley and Sons; 2016.

[36] VanFrank B, Malarcher A, Cornelius ME, Schecter A, Jamal A, Tynan M. Adult smoking cessation–United States, 2022. MMWR Morb Mortal Wkly Rep. 2024;73(29):633-641. doi:10.15585/mmwr.mm7329a1 39052529 PMC11290909

[37] Sánchez-Romero LM, Cadham CJ, Hirschtick JL, . A comparison of tobacco product prevalence by different frequency of use thresholds across three US surveys. BMC Public Health. 2021;21(1):1203. doi:10.1186/s12889-021-11283-w 34162379 PMC8223313

[38] Hawaii Beach Rentals. How many people visit Hawaii each year—tourism statistics. Accessed October 29, 2024. https://www.hawaiianbeachrentals.com/hawaiitravelblog/how-many-people-visit-hawaii-each-year-tourism-statistics/

[39] Barker DC, Wang S, Merriman D, Crosby A, Resnick EA, Chaloupka FJ. Estimating cigarette tax avoidance and evasion: evidence from a national sample of littered packs. Tob Control. 2016;25(suppl 1):i38-i43. doi:10.1136/tobaccocontrol-2016-053012 27697946 PMC5099225

[40] Virginia State Crime Commission. Illegal cigarette trafficking. 2013. Accessed October 29, 2024. https://vscc.virginia.gov/Illegal%20Cigarette%20Trafficking.pdf

