# Supplementary material for: State and Sociodemographic Trends in US Cigarette Smoking With Future Projections
Source: JAMA Netw Open. 2025 Apr 25;8(4):e256834. doi: 10.1001/jamanetworkopen.2025.6834 (PMC12032569; doi:10.1001/jamanetworkopen.2025.6834)
Supplement: Supplement 1. — eFigure. Individual State Models With Projections for States eTable. Parameters Used for Selecting Each State Model With Best ﬁt to Observed Data [file jamanetwopen-e256834-s001.pdf]

Supplemental Online Content

Stone MD, Pierce JP, Dang B, et al. State and sociodemographic trends in US cigarette smoking with future projections. *JAMA Netw Open.* 2025;8(4):e256834. doi:10.1001/jamanetworkopen.2025.6834

eFigure. Individual State Models With Projections for States

eTable. Parameters Used for Selecting Each State Model With Best fit to Observed Data

This supplemental material has been provided by the authors to give readers additional information about their work.

© 2025 Stone MD et al. *JAMA Network Open*.

eFigure. Individual State Models With Projections

A. Tertile 1

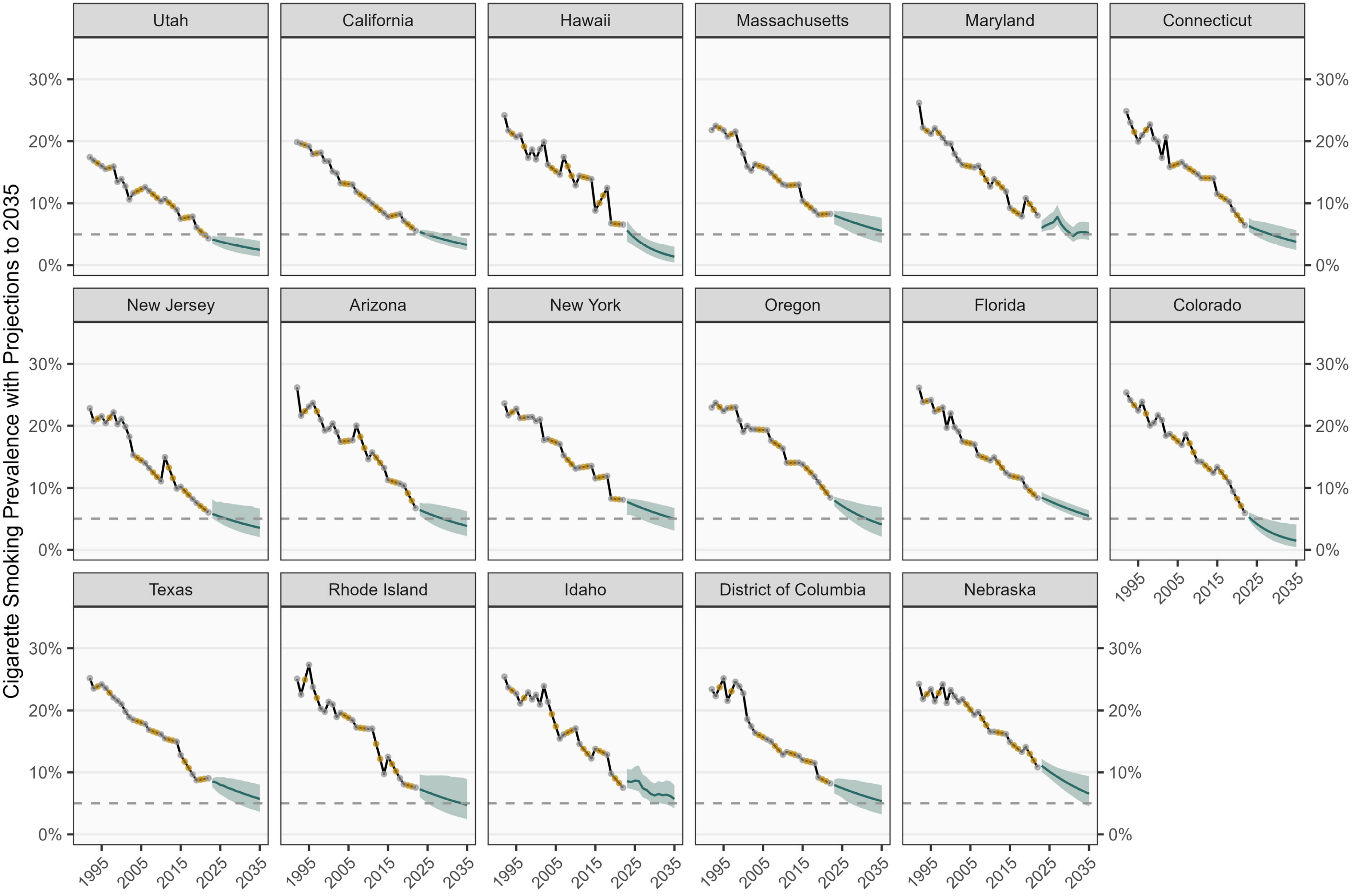

© 2025 Stone MD et al. *JAMA Network Open*.

B. Tertile 2

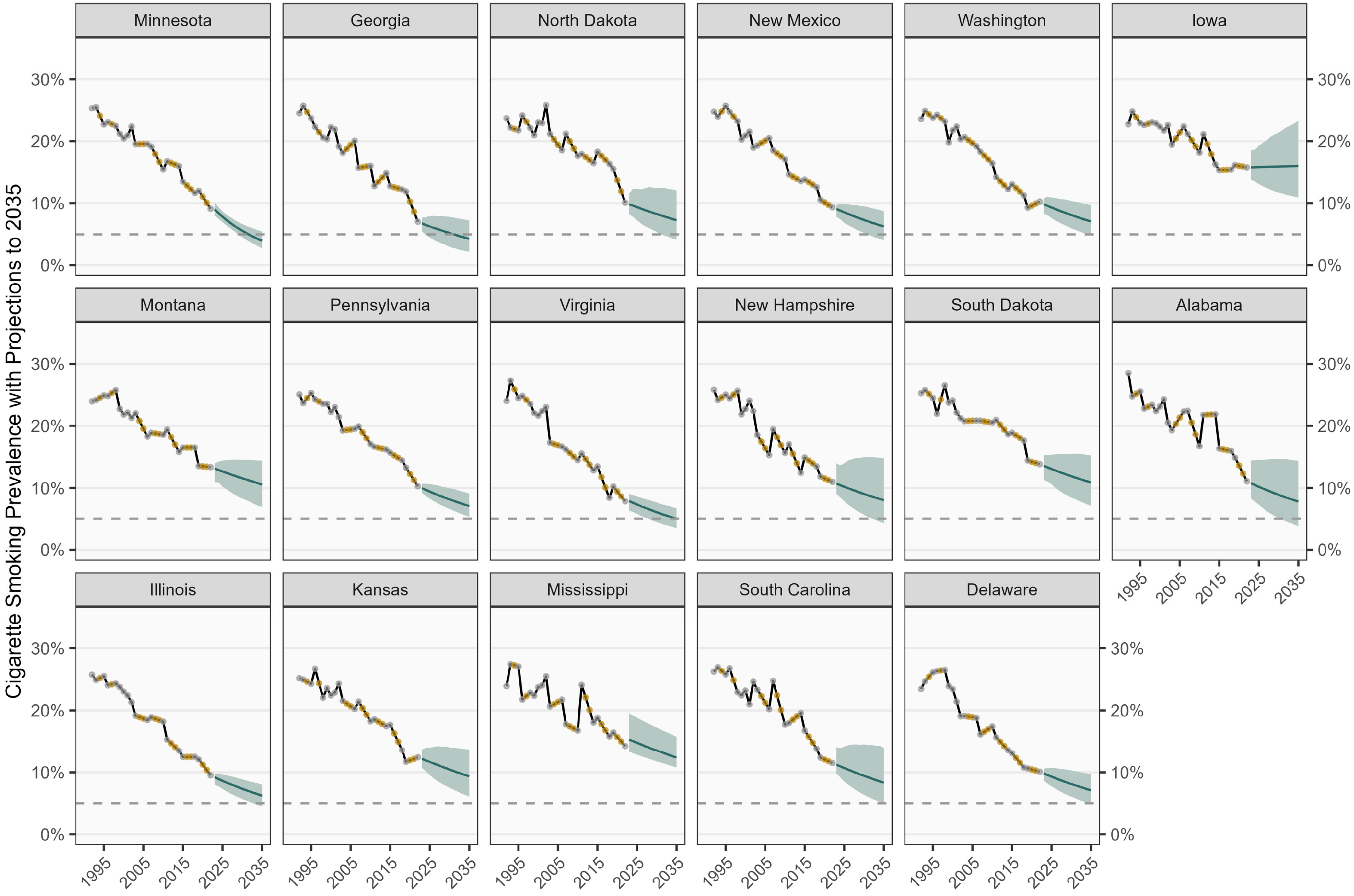

© 2025 Stone MD et al. *JAMA Network Open*.

C. Tertile 3

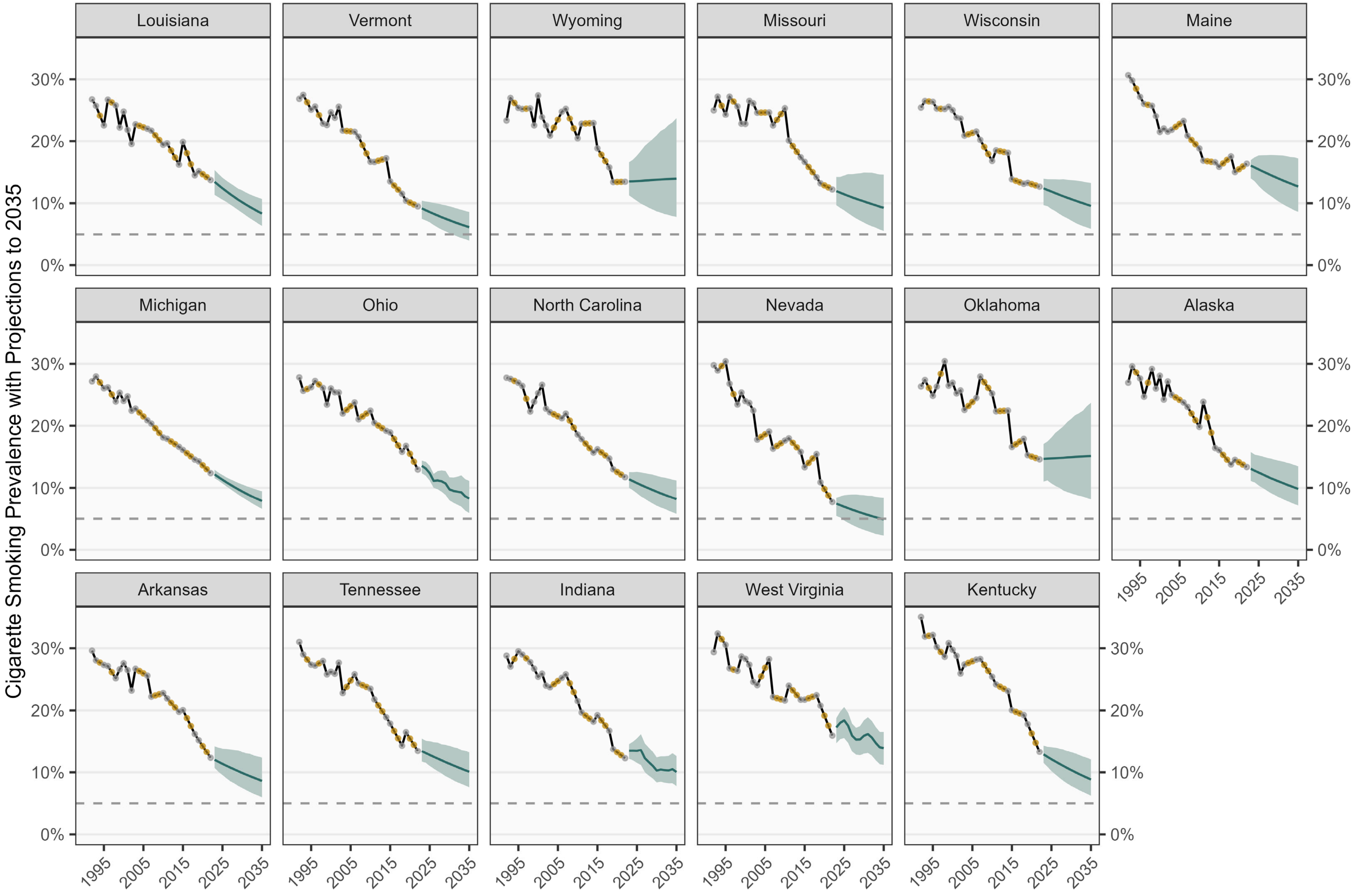

© 2025 Stone MD et al. *JAMA Network Open*.

eTable 1. Parameters used for selecting each state model with best fit to observed data

State	ARIMA		ETS		Selected Model[3]
	Parameters[1]	RMSE	Parameters[2]	RMSE	
Alabama	ARIMA (0,1,0) w/ drift	0.01835	ETS (A,N,N)	0.01941	ARIMA
Alaska	ARIMA (0,1,1) w/ drift	0.01666	ETS (A,N,N)	0.01828	ARIMA
Arizona	ARIMA (0,1,0) w/ drift	0.01249	ETS (A,A,N)	0.01249	ARIMA
Arkansas	ARIMA (0,1,0) w/ drift	0.01222	ETS (A,A,N)	0.01226	ARIMA
California	ARIMA (0,1,0) w/ drift	0.00511	ETS (A,A,N)	0.00509	ETS
Colorado	ARIMA (0,2,1)	0.01066	ETS (A,A,N)	0.01052	ETS
Connecticut	ARIMA (1,1,0) w/ drift	0.01258	ETS (A,A,N)	0.01310	ARIMA
Delaware	ARIMA (0,1,0) w/ drift	0.00959	ETS (A,A,N)	0.00964	ARIMA
District of Columbia	ARIMA (0,1,0) w/ drift	0.01210	ETS (A,N,N)	0.01312	ARIMA
Florida	ARIMA (0,1,1) w/ drift	0.00818	ETS (A,A,N)	0.00808	ETS
Georgia	ARIMA (0,1,0) w/ drift	0.01336	ETS (A,N,N)	0.01440	ARIMA
Hawaii	ARIMA (0,1,1) w/ drift	0.01664	ETS (A,A,N)	0.01600	ETS
Idaho	ARIMA (4,1,0) w/ drift	0.01100	ETS (A,A,N)	0.01289	ARIMA
Illinois	ARIMA (0,1,0) w/ drift	0.00718	ETS (A,A,N)	0.00720	ARIMA
Indiana	ARIMA (4,1,0) w/ drift	0.00808	ETS (A,A,N)	0.00998	ARIMA
Iowa	ARIMA (0,1,0)	0.01184	ETS (A,N,N)	0.01178	ETS
Kansas	ARIMA (0,1,0) w/ drift	0.01141	ETS (A,N,N)	0.01216	ARIMA
Kentucky	ARIMA (0,1,0) w/ drift	0.01161	ETS (A,A,N)	0.01187	ARIMA
Louisiana	ARIMA (0,1,1) w/ drift	0.01490	ETS (A,A,N)	0.01458	ETS
Maine	ARIMA (0,1,0) w/ drift	0.00933	ETS (A,N,N)	0.01074	ARIMA
Maryland	ARIMA (4,1,0) w/ drift	0.00867	ETS (A,A,N)	0.01069	ARIMA
Massachusetts	ARIMA (0,1,0) w/ drift	0.00773	ETS (A,A,N)	0.00773	ARIMA
Michigan	ARIMA (1,2,1)	0.00553	ETS (A,A,N)	0.00545	ETS
Minnesota	ARIMA (0,1,0) w/ drift	0.00912	ETS (A,A,N)	0.00891	ETS
Mississippi	ARIMA (0,1,0)	0.02252	ETS (A,A,N)	0.01958	ETS
Missouri	ARIMA (0,1,0) w/ drift	0.01603	ETS (A,N,N)	0.01652	ARIMA
Montana	ARIMA (0,1,0) w/ drift	0.00957	ETS (A,N,N)	0.01017	ARIMA
Nebraska	ARIMA (0,1,1) w/ drift	0.00985	ETS (A,A,N)	0.00937	ETS
Nevada	ARIMA (0,1,0) w/ drift	0.01566	ETS (A,A,N)	0.01560	ETS
New Hampshire	ARIMA (0,1,0) w/ drift	0.01546	ETS (A,N,N)	0.01633	ARIMA
New Jersey	ARIMA (0,1,0) w/ drift	0.01194	ETS (A,N,N)	0.01316	ARIMA
New Mexico	ARIMA (0,1,0) w/ drift	0.00987	ETS (A,A,N)	0.00988	ARIMA
New York	ARIMA (0,1,0) w/ drift	0.01031	ETS (A,A,N)	0.01005	ETS
North Carolina	ARIMA (0,1,0) w/ drift	0.01035	ETS (A,N,N)	0.01158	ARIMA

© 2025 Stone MD et al. *JAMA Network Open*.

North Dakota	ARIMA (0,1,0) w/ drift	0.01506	ETS (A,N,N)	0.01566	ARIMA
Ohio	ARIMA (4,1,1)	0.00784	ETS (A,A,N)	0.01106	ARIMA
Oklahoma	ARIMA (0,1,0)	0.01885	ETS (A,N,N)	0.01879	ETS
Oregon	ARIMA (0,2,1)	0.00779	ETS (A,A,N)	0.00772	ETS
Pennsylvania	ARIMA (0,1,0) w/ drift	0.00730	ETS (A,A,N)	0.00755	ARIMA
Rhode Island	ARIMA (0,1,0) w/ drift	0.01462	ETS (A,N,N)	0.01575	ARIMA
South Carolina	ARIMA (0,1,0) w/ drift	0.01582	ETS (A,N,N)	0.01659	ARIMA
South Dakota	ARIMA (0,1,0) w/ drift	0.01126	ETS (A,N,N)	0.01188	ARIMA
Tennessee	ARIMA (0,1,0) w/ drift	0.01285	ETS (A,A,N)	0.01224	ETS
Texas	ARIMA (1,1,2) w/ drift	0.00472	ETS (A,A,N)	0.00565	ARIMA
Utah	ARIMA (0,1,0) w/ drift	0.00759	ETS (A,A,N)	0.00762	ARIMA
Vermont	ARIMA (0,1,0) w/ drift	0.01207	ETS (A,A,N)	0.01186	ETS
Virginia	ARIMA (0,1,1) w/ drift	0.01353	ETS (A,A,N)	0.01359	ARIMA
Washington	ARIMA (0,1,0) w/ drift	0.01023	ETS (A,A,N)	0.01001	ETS
West Virginia	ARIMA (4,1,0) w/ drift	0.01236	ETS (A,N,N)	0.01802	ARIMA
Wisconsin	ARIMA (0,1,0) w/ drift	0.01046	ETS (A,N,N)	0.01125	ARIMA
Wyoming	ARIMA (0,1,0)	0.01858	ETS (A,N,N)	0.01848	ETS

Note. ARIMA = Autoregressive Moving Average Algorithm; ETS = Exponential Smoothing Algorithm; RMSE = Root Mean Squared Error. [1]Automatic ARIMA modelling utilized the Hyndman-Khandakar algorithm which combines unit root tests, minimization of the AICc and MLE to obtain the best fitting ARIMA model. [2] Exponential Smoothing is a weighted average model of historical observations, with the weights decaying exponentially as the observations move back further in time. Exponential smoothing has the three main components (Error, Trend, Season) that are each able to be modeled in three different ways (Additive [A], Multiplicative [M], and None/Not included [N]). [3] Model selection minimizing the Corrected Akaike's Information Criterion (AICc) based on the algorithm with the smallest RMSE.

© 2025 Stone MD et al. *JAMA Network Open*.
