# Supplementary material for: State and Sociodemographic Trends in US Cigarette Smoking With Future Projections
Source: JAMA Netw Open. 2025 Apr 25;8(4):e256834. doi: 10.1001/jamanetworkopen.2025.6834 (PMC12032569; doi:10.1001/jamanetworkopen.2025.6834)
Supplement: Supplement 2. — Data Sharing Statement [file jamanetwopen-e256834-s002.pdf]

Data Sharing Statement

Stone. State and Sociodemographic Trends in US Cigarette Smoking with Future Projections. *JAMA Netw Open*. Published April 23, 2025. doi:10.1001/jamanetworkopen.2025.6834

Data
Data available: Yes
Data types: Deidentified participant data, Data dictionary
How to access data: https://cancercontrol.cancer.gov/brp/tcrb/tus-cps
When available: With publication

Supporting Documents
Document types: None

Additional Information
Who can access the data: anyone requesting through public use website
Types of analyses: for any purpose
Mechanisms of data availability: without investigator support
